# Advancing the 3Rs: innovation, implementation, ethics and society

**DOI:** 10.3389/fvets.2023.1185706

**Published:** 2023-06-15

**Authors:** Herwig Grimm, Nikola Biller-Andorno, Thorsten Buch, Maik Dahlhoff, Gail Davies, Christopher R. Cederroth, Otto Maissen, Wilma Lukas, Elisa Passini, Elin Törnqvist, I. Anna S. Olsson, Jenny Sandström

**Affiliations:** ^1^Messerli Research Institute, University of Veterinary Medicine Vienna, Medical University of Vienna, University of Vienna, Vienna, Austria; ^2^Institute of Biomedical Ethics and History of Medicine, University of Zurich, Zurich, Switzerland; ^3^Institute of Laboratory Animal Science, University of Zurich, Zurich, Switzerland; ^4^Institute of in vivo and in vitro Models, Department of Biomedical Sciences, University of Veterinary Medicine Vienna, Vienna, Austria; ^5^Department of Geography, University of Exeter, Exeter, United Kingdom; ^6^Swiss 3Rs Competence Centre, Bern, Switzerland; ^7^Federal Food Safety and Veterinary Office, Animal Welfare Division, Bern, Switzerland; ^8^Innosuisse - Swiss Innovation Agency, Bern, Switzerland; ^9^National Centre for the Replacement, Refinement and Reduction of Animals in Research (NC3Rs), London, United Kingdom; ^10^Department of Animal Health and Antimicrobial Strategies, Swedish National Veterinary Institute (SVA), Uppsala, Sweden; ^11^Institute of Environmental Medicine, Karolinska Institutet, Solna, Sweden; ^12^Laboratory Animal Science, i3S-Instituto de Investigação e Inovação em Saúde, Universidade do Porto, Porto, Portugal

**Keywords:** animal research, 3Rs, innovation, implementation, ethics

## Abstract

The 3Rs principle of replacing, reducing and refining the use of animals in science has been gaining widespread support in the international research community and appears in transnational legislation such as the European Directive 2010/63/EU, a number of national legislative frameworks like in Switzerland and the UK, and other rules and guidance in place in countries around the world. At the same time, progress in technical and biomedical research, along with the changing status of animals in many societies, challenges the view of the 3Rs principle as a sufficient and effective approach to the moral challenges set by animal use in research. Given this growing awareness of our moral responsibilities to animals, the aim of this paper is to address the question: Can the 3Rs, as a policy instrument for science and research, still guide the morally acceptable use of animals for scientific purposes, and if so, how? The fact that the increased availability of alternatives to animal models has not correlated inversely with a decrease in the number of animals used in research has led to public and political calls for more radical action. However, a focus on the simple measure of total animal numbers distracts from the need for a more nuanced understanding of how the 3Rs principle can have a genuine influence as a guiding instrument in research and testing. Hence, we focus on three core dimensions of the 3Rs in contemporary research: (1) What scientific *innovations* are needed to advance the goals of the 3Rs? (2) What can be done to facilitate the *implementation* of existing and new 3R methods? (3) Do the 3Rs still offer an adequate ethical framework given the increasing *social awareness* of animal needs and human moral responsibilities? By answering these questions, we will identify core perspectives in the debate over the advancement of the 3Rs.

## 1. Introduction

The 3Rs principle is recognized in many different places where animals are used in research (1). Since their original formulation in 1959 in *The Principles of Humane Experimental Technique*, the 3Rs have become widely accepted as a prerequisite of responsible, high-quality science in which adequate ethical consideration is given to the human use of animals (2). However, it was not until the 1990s that they were translated into policies that could be used to regulate the practice of using animal models in research (2, 3). The 3Rs are now incorporated as legal requirements in transnational legislation such as the European Directive 2010/63/EU (4) as well as in a number of pieces of national legislation, including some in Switzerland and the UK.

The European Directive 2010/63/EU (4) (henceforth: the Directive) on the use of animals for scientific purposes is arguably internationally the most far-reaching piece of laboratory animal legislation and is often referred to as the most extensive animal experimentation legislation in the world. While the USA, China and Japan use more animals than the European Union (EU) (5), their national legislation protecting animals used in research is more limited. However, legislation that resembles the Directive in large parts and detail can also be found in non-member states like Switzerland [(6), pp. 305–325].

The use of animals for scientific purposes is lawful in EU Member States if all the of the conditions – including the 3Rs principle – set out in the Directive and the corresponding national acts are met. The Directive also contains more overarching goals, like respecting the intrinsic value of animals and promoting public awareness of our ethical responsibilities to them (Directive, recital 12), and it speaks of the need to work toward full replacement of live animals in experiments (Directive, recital 10). Hence, it navigates between protecting animals, on the one hand, and allowing their use in research, on the other. In this respect, the 3Rs principle, together with harm-benefit analysis (Art. 38 Directive), provide a nexus for the different values embodied in the Directive: the principle and analysis hold things together that are otherwise in tension – namely, the value intrinsic to animals and the value of scientific benefits such as knowledge gain, safety and education.

It is not surprising that advocates at both ends of the spectrum – i.e. those against animal use in research and those in favor of it – challenge the 3Rs principle: if replacement is possible, why do we still use animals, and is the 3Rs principle not stabilizing the status quo (7, 8)? Indeed, does the 3Rs principle really have the potential to lead the way to animal-free research at all? Such questions have also reached the political arena. To name a few: In 2015 the European Citizens’ Initiative *Stop Vivisection* gathered the necessary support to present the demand to the European Parliament to phase out animal research (9). In 2021, the European Citizens’ Initiative *Save Cruelty Free Cosmetics* was issued and supported by more than 1.2mio signatories (10). In 2022, the *FDA Modernization Act 2.0* was signed by President Biden that allows for alternatives to animal testing for purposes of drug and biological product applications (11). In early 2022, 2.4 million Swiss citizens voted on a proposal to ban animal (and human) research in Switzerland. The plebiscite resulted in 21% in favor and 79% against the proposition (12). Although neither of these initiatives and legal changes led to a ban of animal use for scientific purposes, they indicate that its acceptance cannot be taken for granted.

In contrast to positions that view animal-free research as an urgent priority, others have argued that we risk losing relevant and important gains in knowledge by refraining from using animals in research (13–15). Debates range widely across ethical positions, whereas the legal context is clear: animals can lawfully be used in research if the conditions of the Directive and implemental legislation in Member States are met, and this requires compliance with the 3Rs principle [Directive, Art. 38 (2) b].

Nevertheless, commentary and debate continue to revolve around how far the 3Rs operate in ways that are sufficient to manage the ethical complexities of animal use in research (16, 17) at the forefront of scientific fields (18) and as social priorities diversify and change [(19); recital 12 Directive]. These debates are increasingly informed by work from the social sciences and humanities looking in detail at the intersection of the social, scientific and ethical processes through which the 3Rs emerged and are applied today (20, 21). Hence, the aim of this paper is to reflect on the structure of this complex arena in an effort to show more clearly where the principle of the 3Rs has potential and limits as regards its innovation and implementation. Furthermore, we will touch upon the complexities of related normative questions and the issue of whether the 3Rs principle remains timely and appropriate as an ethical framework, given the increasing social awareness of animal needs and human moral responsibilities.

This brief introduction may already teach us some critical things about the debate on animal use in research and the 3Rs. First, the 3Rs principle offers guidance in a system of regulation in which animal research is legally permitted but must be justified. Second, observance of the 3Rs principle is an important element of responsible research practice within the given legal framework, but it is not a way to change the existing legal foundation. Hence, third, there is discord between the view that the 3Rs principle allows only for minimal change and may even sustain the status quo rather than leading to desirable, more radical, change.

Here, we address the 3Rs principle’s potential, limitations and complexity as a policy instrument in the service of advancing science and knowledge as well as protecting animals. Hence, we will describe successes and challenges of the 3Rs by looking into the past, but we will also consider developments in the innovation, implementation (in the sense of changing practice) and understanding of the 3Rs. Finally, we will contextualize the 3Rs principle in the broader societal debate and sketch some of its limits and short comings as an ethical framework.

## 2. The (missing) 3R effect: who is missing what?

At the same time as the 3Rs principle has been gaining influence via its integration into legislative frameworks across the world, its effectiveness is coming under growing scrutiny by various stakeholder groups (22). In particular, the discussion of why implementation of the principle has not progressed further is on the table, as is debate over why its implementation has not had a “stronger” impact on, for example, the number of animals still being used in research [(23–27); Expectations concerning measurable effects of the 3Rs principle are high, both from political and from public perspectives. The fact that there has been no substantial and consistent decrease in the absolute number of animals being used in experiments [e.g., (28); for difficulties of comparing the numbers of animals used in the EU see: (29)] is perceived as a “missing” 3Rs effect. However, to understand how the 3Rs principle can have a genuine influence as a guiding instrument we need to look beyond the simple measure of total numbers. As we will see later in this article, quantifying the effect of any one of the 3Rs is a challenging task in itself. Of course, the scarcity of measurement tools need not prevent us from working toward ensuring the principle has a greater impact. This article will give some selected examples of successful advances in the application of the 3Rs, as well as pointing to some areas where the authors see ways to drive 3Rs implementation further.

**Figure 1:**
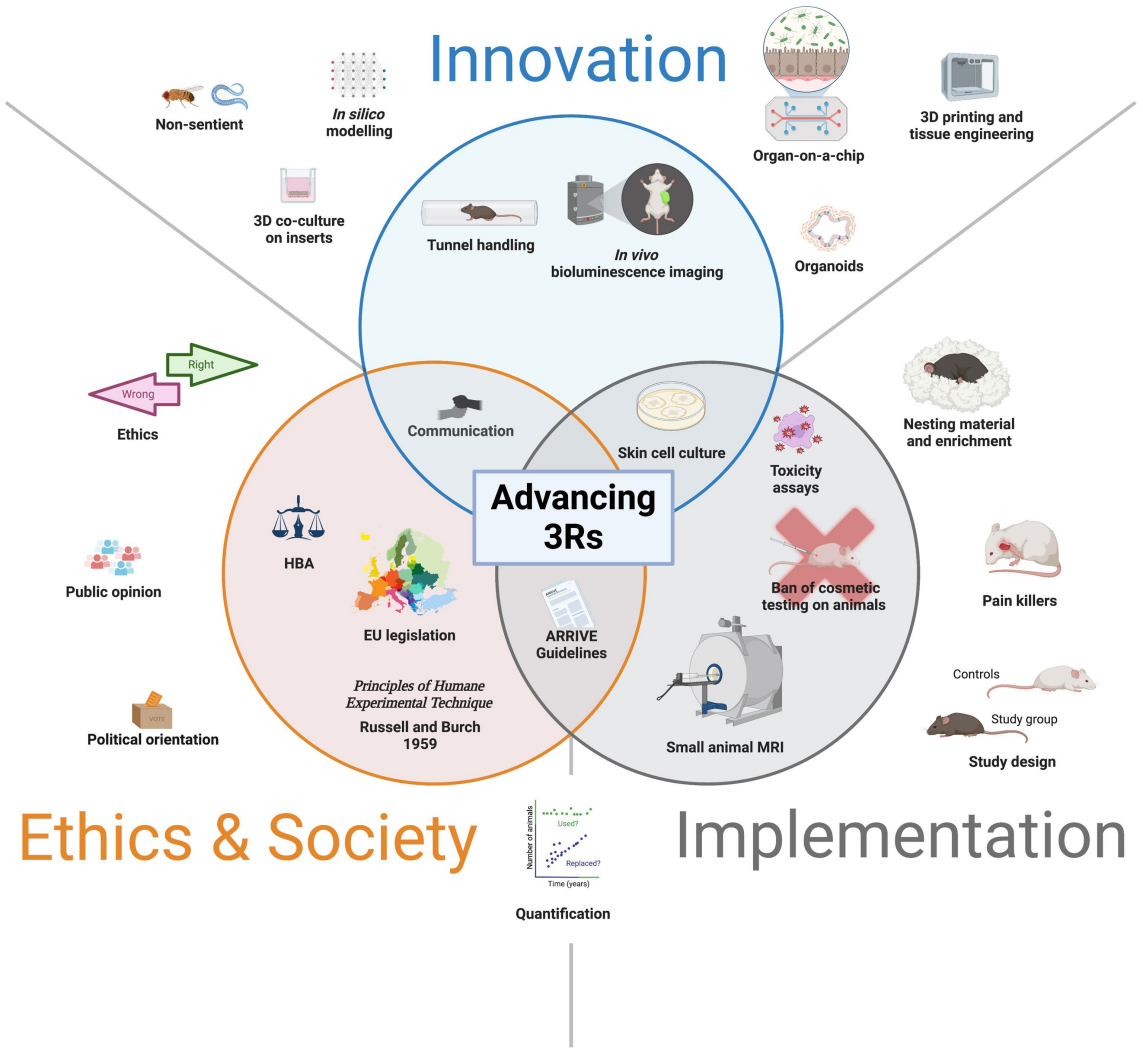
The complexities of advancing 3Rs: Innovation, implementation and societal issues as interrelated core dimension. Advancing 3Rs. This schematic illustrates the intersection of innovation (blue), implementation (grey) and ethics & society (orange), all directed toward advancing 3Rs. Concerning innovation, the recent development of non-aversive methods for manipulating rodents such as tunnel handling have provided major improvements in the *refinement* of animal welfare, whereas and *in vivo* bioluminescence imaging has enabled the long-term non-invasive monitoring of animals leading to a *reduction* of their use for research purposes. As innovation is directly causal to implementation, the development of skin cell cultures for the *replacement* of *in vivo* testing, has led to validated and approved toxicity assays, followed by the ban of cosmetic testing on animals. Likewise, imaging techniques such as MRI have been adapted to small animals and helped assessing organ function in living animals. Lying at the conference of ethics and implementation are the ARRIVE guidelines that have been developed to improve research reporting and quality and minimize the waste of animals used in research. Within ethics and society, since the publication of Russell and Burch in 1959 on the 3Rs principle, its incorporation within the legislation has contributed to the development of the harm and benefit analysis (HBA) in ethical committees that legislate the use of animals in experimentation. From innovation toward ethics & society, as the science goes public, there is an increased involvement of the society in the ethical debate and political decisions regarding the use of animals in research. In the periphery are shown all the domains where challenges are found as for instance in innovation (e.g., 3D co-culture or organs-on-a-chip), implementation (e.g., use of pain killers in surgical practice or enrichment for improved animal welfare), and in ethics & society (e.g., harmonized EU legislation and the contribution of the public opinion), all of which should be addressed for generating a responsible scientific knowledge.

We propose that there are three core dimensions where more knowledge and understanding is needed to increase the impact of the 3Rs principle: What scientific *innovations* are on the horizon that will contribute to the goals of the 3Rs? How can existing and new 3R methods be *implemented* to greater effect? Linking the principle back to the societal debate: In what ways does the 3Rs principle embrace *societal expectations* about our moral responsibilities to animals? From this angle, improved effects of the 3Rs might be found not only on the scientific level, in terms of method development and implementation, but also on the institutional, legal, societal and ethical levels, in terms of reconsidering and developing the moral ideals contained in the 3Rs principle and corresponding practice. These three dimensions (roughly speaking: innovation, implementation, ethics/societal matters) are naturally interdependent. They anchor the perspectives laid out below and guided the development of the Swiss National Research Programme 79 “Advancing 3R – Animals, Research and Society” that has been launched in 2021 by the Swiss National Science Foundation (SNSF) (Figure 1).

## 3. Advancing 3Rs innovation

### 3.1. Successes and developments in innovation

Innovation is key to ensuring that the effects of the 3Rs become stronger by providing new research methods. Innovation in the replacement of animals in research, either entirely or at least partly, enjoys a special status from an ethics perspective in that it addresses the key problem that animals are instrumentalised: they are used to gain knowledge at the cost of their suffering (30). Historically, most of the 3R research funding, by some distance, has supported innovation in replacement, largely as part of the effort to move toward animal-free toxicology and safety testing (referred to as New Approach Methodologies, NAMs) [e.g., (31, 32)].

Innovative replacement tools and methods that allow the scientific objective to be achieved without animal use include novel cell culture approaches (e.g., tissue culture, organoids, organs-on-chip and microphysiological systems), micro-dosing, non-sentient animals, *in silico* modeling and many other techniques. Yet, innovation is equally important for reduction and refinement, by facilitating research that relies on fewer animals and causes less harm to those that are used. Three examples of successful 3Rs innovation are: the development and validation of *in vitro* methods of hazard and safety testing of cosmetics, replacing the existing animal tests (33, 34); imaging techniques making it possible to follow change in tumor size or bacterial load within the same animal over a period of time (instead of euthanising and dissecting a cohort of animals for each timepoint) as a means of animal use reduction (35); and the introduction of tunnel handling of mice as a refinement technique (36).

To understand how to strengthen the effect of the 3Rs it is worth considering how the contexts in which these innovations have emerged differ. While the driver of the development of alternatives to animal-based testing of cosmetics was largely political, innovation developed in bioengineering was necessary for the successful replacement of the traditional tests. Deep understanding of the mechanisms of skin sensitisation laid the foundation for a remarkable technical development in *in vitro* approaches (37) that has presented us with unparalleled opportunities to assess human health outcomes without using animals. The political decision, in the early 1990s, to ban cosmetics testing on animals in the EU was further accompanied by investment in research into alternatives. That was important because the ban was conditional upon there being validated alternatives that could be implemented. This can be described as a top-down approach, because policy decisions drove innovation.

In contrast, the new handling methods for mice are an example of innovation changing established practice from the bottom upwards. The first paper on tunnel and cup handling methods by Hurst and West (36) built on research partly funded by the UK’s National Centre for the Replacement, Refinement and Reduction of Animal Research (38) through a response mode funding call. The paper reported strong and convincing results showing that tail handling (until recently the predominant method) resulted in more anxious mice – mice that never habituated to the handling method – in comparison with the less aversive and innovative tunnel and cup handling methods. The results attracted great interest in the laboratory animal science community and were especially important for animal technicians who were handling mice in their daily activities. Their dissemination has also been facilitated by the NC3Rs. But the starting point was the innovation, in a bottom-up approach where it was the grant proponent’s particular research interest that motivated the choice of topic. Of course, a dynamic combination of top-down and bottom-up approaches is often at work, and industry development may also play a key role, as happened in the adaptation of already existing biomedical imaging techniques for use in small laboratory rodents.

### 3.2. Complexities: testing required by regulation vs. hypothesis-driven research

Innovation in replacing animal methods in *testing required by regulation* stands out in several ways that are relevant in the discussion. Safety and hazard testing contrast starkly with investigative research: there is a high level of standardization when testing is a strictly regulated process with clearly defined internationally approved guidelines introduced by the Organization for Economic Co-operation and Development (OECD). This makes it comparatively easy to determine clear innovation needs in safety testing (e.g., to identify a particular test that needs to be replaced) and above all to determine when the needs have been met.

Whereas in context of the OECD test guideline program non-animal methods can observably replace previous animal use, this contrasts with, for example, biomedical and disease research, where there is no standardization of the approaches, where the process may be distributed over many laboratories in many institutions, and where researchers’ work is driven by many different research interests. Experimental biomedical research is conducted using a combination of methods, including *in silico*, less complex *in vitro* (e.g., 2D cell cultures) and more complex *in vitro* (e.g., organoids, organ-on-a-chip) techniques, plus animal experiments. In scientific research, the same method can be used for many different purposes, and the same purpose can be achieved by many different methods. Hence, alternative methods are not necessarily seen by researchers as replacement methods, but rather as new-approach methods (NAMs). In this regard, a terminological clarification is in order: “New-approach methods” is not the same as “non-animal methods.” Non-animal methods, if used as alternatives to animal models, are” non-animal alternatives,” in which case they indeed function as replacement. However, NAMs are often not developed with a direct replacement in mind; instead, they are rather a different way to approach a research question. Hence, they may not be developed primarily with the intention of replacing a specific animal model. This means that innovations around alternatives in biomedical research are often highly specific; furthermore, the detailed information on methods, materials and data that would help evaluate their use in other situations is not always reported (39).

### 3.3. Refinement: replacement’s “poor cousin”?

Although in principle all the 3Rs have the same status, in practice refinement often comes across as the “poor cousin” of the other Rs. In comparison with replacement that goes hand in hand with the potential to reduce the number of animals used in many instances, both funding and the positioning of the research field are constraints. For many years, there was little to no public investment in any 3Rs research with the exception of targeted funding for replacement in animal testing. The first public funding to be available for all of the 3Rs was provided in Switzerland in 1987. Judging by the 146 projects funded over the 35-year period since, there has been a clear tendency to fund replacement-focused projects rather than refinement projects (40). Substantial funding for refinement research was arguably not provided by any funding agency until the establishment of the NC3Rs in the UK in 2004, then with an annual budget of £700,000 to be distributed across all of the three Rs (41). For comparison, the year before, the European Commission (42) reported that it had already spent €63 million on research into non-animal alternatives.

To date, much of the innovation in replacement has involved tissue engineering and other advances in cell culture techniques, research topics that are centrally positioned in both biomedical and bioengineering research. Refinement straddles veterinary medicine and the specific biomedical research discipline in question, and for both disciplines, refinement-related research questions tend to be peripheral. In veterinary medicine, laboratory animal science is a small field, sometimes seen as a low-priority research area. Likewise, biomedical refinement research, and other methodology-related research, is generally considered secondary in the research community where animals are used. The problem is exacerbated by the fact that successful refinement research often requires collaboration between experts in animal welfare and highly specialized scientists in a biomedical discipline. For example, better pain control would refine many animal experiments, but whether a pain treatment affects the results of an experiment can only be properly assessed by the specialised scientists (43). Generally speaking, that target can only be achieved with systematic research of species-specific, model-specific and non-model-specific refinement needs. Otherwise, attempts to implement refinement will be limited to best veterinary practice and collective practical wisdom, often relying on scientifically unproven assumptions. Funding schemes addressing refinement play a crucial role in providing context and support for the kind of collaborative research that is required to develop and validate innovative refinement measures. Hence, when substantial funding for refinement research is put in place, biomedical researchers will be more interested in, and likely to pursue, this kind of research and then feed new knowledge into a culture of care.

In short, refinement is seen as a non-instrumental part of the process of generating data, and therefore the innovative refinement of experimental approaches – e.g. creating less invasive ways of administrating substances (44) or earlier and less severe endpoints (45) – will rarely be driven by research groups that use animals in experimental settings. Often, this is not a kind of research that is considered suitable for the main journals in a given field of research, and this very fact will act as an obstacle for young biomedical researchers when considering where to focus their early-career effort. This means that the group of researchers from which successful bottom-up proposals (e.g., picking mice up using refined methods (36)) can be expected will be very small, unless refinement research is given greater priority and higher status.

Innovation in refinement has suffered from this situation (with the possible exception of refinement innovation in industry, where many pharmaceutical companies set aside budgeting and time for smaller refinement projects), and there are clear gaps that need addressing. Moreover, although refinement comes across as the poor cousin of the other Rs in funding, on the practical level of project evaluation for authorization of experiments with animals, refinement seems to be attended to at the expense of in particular replacement; a problem identified already 20 years ago in the Swedish context (46). Replacement requests from an ethics committee require highly specialized expertise in the research field in question, which the committee is unlikely to have. This might shift the focus of the committee work on refinement, which again highlights the need for reliable methods to quantify animal welfare. Hence, the development of knowledge about the interaction of animal welfare and biomedical research is crucial for reducing the negative impact of research and committee work.

A recent systematic review shows the considerable heterogeneity of measures to assess animal welfare in response to environmental enrichment, noting that this heterogeneity makes it impossible to perform reliable meta-analyses (47). Recent tools in fish, relying on an automated monitoring of behavior (48), have been able to capture the impact of post-operative analgesia after fin-clipping on behavioral patterns, thus allowing inferences to be made on the benefits on animal welfare (49). Similar technologies of home cage monitoring for mice have been able to detect the benefits of environmental enrichment against aggression, thus overall improving welfare (50). Such tools may allow indirect objective assessment of animal welfare in a general manner, offering greater applicability such as for assessing pain in animals or evaluating the animal welfare impact of different euthanasia methods.

### 3.4. Conclusions on advancing 3Rs’ innovation

To summarize, innovation has contributed to the impact of 3Rs in the past and will continue to be crucial. Cosmetics testing is an example of how far innovation can take us when it is based on already established biological knowledge (e.g., key events that indicate likelihood of skin sensitization being provoked by a given substance) and technical advances (e.g., biologically non-reactive materials for cell cultures) that allow the innovation to be translated into an effective method of replacement in cosmetic testing and the testing of industrial chemicals. While they are an impressive achievement, the *in vitro* systems for cosmetics testing are comparatively simple, in that they represent a single type of a mainly two-dimensional organ. Current innovations in replacement focus on more complex systems with a more pronounced three-dimensional structure (organoids and spheroids) and organ-organ interactions (on-a-chip systems).

## 4. Advancing the 3Rs’ implementation

### 4.1. Successes and developments in implementation

To gain further insights into the effective implementation of the 3Rs, we need to know to what extent animal models are being replaced, animal numbers reduced, and procedures on animals refined. Hence, we need to be able to measure the impact and effects of the 3Rs in some way. Although it is often questioned whether there really are widespread, measurable effects of 3Rs implementation, there has been considerable progress here. Perhaps the most well-known example of a successfully implemented replacement approach is in the integrated testing strategies for assessing the hazard and potency of the skin sensitization in cosmetics discussed above. This effectively allowed animal testing for cosmetics to be banned. What sets this example apart, in terms of implementation, is not only the fact that the biological and technical knowledge was advanced enough to permit the development of the required tests, but also the defined tests themselves (51). Together, these have ensured there is wide acceptance of the relevant regulations allowing for the effective implementation of a political decision.

Replacement strategies can effectively reduce animal numbers – as is very clearly evidenced in the case of cosmetics testing. Similarly, over the past decade, technical developments in *in vivo* small animal imaging have opened up new and improved avenues toward substantial reductions in animal use, while simultaneously allowing more data to be obtained from animals through scientific investigation (52, 53). Other effective reduction approaches have combined several aspects: improved study design, developments in method, and study coordination have together reduced animal use in pharmaceutical toxicity testing (54). The Törnqvist et al. (54) study describes significant animal reductions in both regulatory and investigative safety studies. It concludes that, when they are coordinated at a strategic level, combinations of *in silico, in vitro* and *in vivo* methods effectively contribute to reduction.

Regarding refinement, some measures, such as the use of anesthesia in potentially painful procedures (54, 55) and the provision of nesting material to laboratory rodents, are now standard (and legally required) in the EU. These measures have far-reaching consequences, as indicated in a recent systematic review and meta-analysis in which Cait et al. (56) demonstrate lower rates of morbidity and mortality across a number of animal disease models. However, larger scale attempts to quantify both the implementation and the impact of refinement are generally lacking. For quantitative measures of the implementation of refinement we have to turn to research studies such as systematic reviews of the way refinement is reported in research papers (57, 58) and surveys of researchers and technicians asking about their refinement practice (59, 60).

### 4.2. Complexities of implementation: overcoming barriers

All examples of successful implementation of new 3Rs approaches show that the success is dependent on innovation. Challenges to implementation itself include scientific barriers, such as the limitations of a given method; legal barriers, such as the “gold standard” of animal use in regulatory testing; economic barriers, such as the financial costs and time resources for implementing new approaches; cultural and institutional obstacles, such as difficulties establishing evaluation criteria for academic success that reward 3Rs implementation; and difficulties changing the established mindset. Ongoing efforts to address these issues [e.g., (61–64)] are being made, but the examples are still rare. There are also barriers at the individual and laboratory level, where animal research is sometimes still seen as the nimbus of “serious research.” All of this together creates a rather inert system in which change is difficult.

Hence, the application of the 3Rs principle may be seen as a tiered approach to the choice of method that is linked to research projects generally conceptualized as being primarily driven by hypothesis testing. Here, the model and methods are chosen with reference to the question to be addressed. This points to a disparity in applications of the 3Rs principle to meet legal requirements imposed by animal welfare legislation in EU Member States and European countries like Switzerland. The 3Rs principle need only be considered when a research project applies for a license in a country where that principle is part of the legal framework. Compliance with the 3Rs throughout the research process, from hypothesis to publication, via an experimental planning and analytic phase, is voluntary and depends on awareness of the 3Rs at an organizational as well as individual level (65).

### 4.3. Assessing 3Rs implementation and hurdles

To assess the effectiveness of the 3Rs principle as a policy instrument for advancing humane animal experimentation, appropriate parameters for measuring the effects of the 3Rs are still needed. This becomes evident when we are trying to understand the extent to which, for example, replacement methods have been genuinely implemented. To illustrate this point, we would like to highlight two sides to the problem of assessing replacement’s implementation. First, the metrics track the numbers of animals used rather than those replaced, and second, most replacement approaches are not recognized as such. Thus, assessing the current impact of already implemented replacement methods is highly complex (as it is in toxicology) and may be impossible, as things stand, in some research fields. Similar observations can be made about the assessment of impacts of the implementation of reduction and refinement.

Besides these fundamental challenges in understanding the impact of active utilization of 3R measures, there are practical hurdles to 3Rs implementation. Establishing and validating new methods may be technically very demanding and time-consuming, creating real problems for researchers seeking to implement new techniques in their laboratories. Comparisons with historical data may require the continued use of established methods, and this too may hamper the switch from established *in vivo* methods to new methods. Moreover, to obtain funding researchers might need to prove they have previous experience and skills in the methods chosen for the project, which may further hinder progression in the implementation of alternative approaches.

Moreover, new methods very often (and perhaps most frequently) serve as measures of reduction, by replacing or omitting steps, or elements, of an existing investigative approach. Therefore, the relevance and reproducibility of these new methods can raise further issues when the capacity of a given model to reduce animal use is determined by its *in vitro*-to-*in vivo* translational value. Hence, other scientists or reviewers might ask for the biological relevance to be proven *in vivo* in confirmative studies, which limits the primary reduction effect of replacement methods in such cases.

Rigorous experimental planning that preserves internal and external validity (66–68) – qualities determined by how rigorously a study is performed, and how reliably its results can be applied to other situations (69) – will facilitate strategies reducing or even eliminating the need for confirmatory animal studies. Integrated strategies deploying multiple alternative approaches in combination – as happened in the previously mentioned strategy for skin sensitisation testing of cosmetic products – will be required if we are to extend the implementation of replacement.

Turning to the implementation of refinement approaches, there may be gaps here in researchers’ and license review committees’ awareness of suitable new approaches. Equally obstructive may be a lack, or shortage, of the competencies needed to apply such methods. Applying new methods of experimentation may also jeopardize the academic success of a project, or its productivity, ensuring there is only a modest incentive for the researchers to alter their approach. This applies equally to the implementation of replacement approaches where the required techniques need to be established in a laboratory currently lacking mastery of them. Again, the way in which refinement methods were implemented may not be described in the methodology of scientific articles, which creates difficulties not only in quantifying to what extent such methods have been applied, but also in measuring their potential impact on animal welfare. Therefore, we are again faced with the point that to measure the 3Rs’ effectiveness it is necessary to establish better outcome parameters and their measurements. Given all these complexities, the animal welfare representatives in research facilities can play a crucial role. With their direct contact with the animals and the facility, they are well placed to identify refinement needs and to measure impacts on the animals. If they are provided with appropriate support, they can take a key role in the implementation of measures – e.g. by giving advice on the care and use of the animals, and developing and disseminating local animal welfare policies and standards.

Aside from the challenges inherent in the implementation of effective 3Rs methods in the research process, a number of external factors, both positive and negative, may be exerting influence. For instance, within certain domains, such as pharmaceutical safety testing and regulatory risk assessment, the validation process is established and accepted. Standard *in vivo* tests remain within the legal guidelines and thus determine regulatory acceptance of newly established methods (70). An equivalent formal process of validation is not presently used in academic research and disease models. Moreover, academic success is measured mostly in publications in high-impact journals, and these journals make increasing demands regarding the comprehensiveness of the data, which means that *in vitro* studies alone will often not be considered sufficient (71). Thus, *in vivo* studies can be required as a complement to alternative approaches to improve the validity of the results. More generally, the ever-increasing amounts of data required for a single study may make it even more complex and difficult to implement the 3Rs without impacting knowledge gain or publication success. Challenges to this paradigm in the fields of preclinical research and toxicity/safety testing have recently appeared in the literature (72, 73). “The question is raised, whether continuing to require results of animal testing for publications or grant funding still makes scientific or ethical sense and if more physiologically relevant human Organ Chip models might better serve this purpose” (72). This even opens new avenues leading to the questioning of animal studies as the gold standard in other fields as well.

### 4.4. Conclusions on advancing 3Rs’ implementation

In summary, if 3R measures are not implemented, there can be no 3R effects. Additionally, assessing the effects of successfully implemented approaches is far from straightforward given that appropriate outcome parameters and measurement tools are largely missing for replacement, reduction and refinement alike. The only easily accessible parameter of progress in the implementation of the 3Rs we have is one recording those methods that have satisfied regulatory requirements or have been included in national or international regulation: examples are validated alternative tests that replace specific toxicity/safety tests and requirements in environmental enrichment. The total number of animals used in research is often referred to as an outcome measure, but this is highly problematic. At a national level, total numbers depend on many factors, including research activity, the level of investment into research and development, coherent data collection, and so on. Also, they are often presented without correction for the number of biomedical researchers in the respective country at any given time point. Further, changes in reporting and, for example, (severity) classification are usually not reflected in these statistics.

For a nuanced understanding of the effect of the 3Rs it is necessary to generate suitable information through, for example, systematic reviews or questionnaire studies – a kind of 3R “meta-research” that itself needs adequate funding. Hurdles to 3Rs implementation lie at various levels, moreover. They include difficulties in changing established practices, institutional obstacles, and limited awareness and resources, to mention just some. Although the complexity is immense, pragmatic solutions to overcome these hurdles are within reach if the relevant incentives are put in place.

## 5. Advancing 3Rs: ethics and society

### 5.1. The 3Rs’ development and translation in theory and practice

In the course of scientific developments and changes in human-animal relations in societies, the 3Rs principle has proved adaptive in various respects. One way to respond to new demands in the debate on the use of animals in research was to add complementary Rs, such as a fourth R for Responsibility (74–76), or Rs designed to enhance scientific value by adding, for example, Robustness, Registration or Reporting (77). Further Rs can be found in the literature, including Replication, Reproducibility and Rigor. There has even been talk of a “Rhumba of Rs” (77, 78). Such additions can be read as a reaction to developments and new issues in science and society that challenge both the breadth of applicability and sufficiency of the original 3Rs principle.

We propose that ongoing ethical and social challenges around the 3Rs can be understood by looking at four ways in which the 3Rs have come into widespread use via a set of active and often local translations. First, the moral foundation of the 3Rs has been the subject of academic debate for the past few decades (79). This foundation merits ongoing scrutiny, as the normative and empirical premises underwriting the moral status of animals and their relationship to humans continue to evolve. Considering this, animal research ethics has developed into a recognized subfield of applied ethics (80, 81). Second, there is the translation from ethical principles into practical recommendations. Russell and Burch (82) first articulated this in the late 1950s, but questions remain, in interdisciplinary debates, around who should define the 3Rs: in societies, professional bodies, campaigning organizations and academic communities today (22, 83, 84). Third, there is the translation of recommendations into national research regulations. Proposals on how to translate the 3Rs into regulations reflect ideas about the proper role of the state in limiting academic freedom and promoting research innovation, as well as in governing scientific procedures and protecting animals (85). Finally, there is the translation of regulatory requirements built upon the 3Rs into everyday policy and scientific practice – from animal technicians seeking to refine procedures (86), to scientists imagining public attitudes when deciding which species to use (87), or institutions creating barriers to the use of replacements (88). There is also the translation of the whole 3Rs endeavor into the public sphere in order to raise awareness and gain support through transparent communication (89). For Russell and Burch, writing within the academic culture of 1950s Oxford, this translation into the public sphere was imagined as something that would come at the end of the process. Today, moves toward openness and transparency in science, together with the growing agenda of responsible innovation (90), mean that the vantage points from which the public may challenge animal research have multiplied. This implies a need for ongoing review around how far the 3Rs principle meets its intended ethical purpose.

The 3Rs principle is not a strategy for phasing out animal use in research. In this respect, the 3Rs principle seems to be in line with the general public’s majority view. For instance, in a Danish empirical survey (91) 30–35% of people questioned approved of animal research quite strongly, and 15–20% opposed animal research, whereas the remaining 50% were reserved in their views. In a Swedish survey (92) just over half (55%) of respondents considered that animal experiments are acceptable in medical research. Acceptance increased to 82% when it was stipulated that the animals were being treated well and not exposed to unnecessary suffering (92). In the UK, overall, the public (i.e., British adults aged 15+) is supportive of the use of animals in scientific research: 68% agreed in 2014 that it is acceptable “so long as it is for medical research purposes and there is no alternative,” but there is also widespread agreement (76%) that more work should be done to find alternatives to using animals in such research [(93); for the UK, see also Ipsos Mori (94) and for the U.S. Gallup (95)]. A recent qualitative study conducted in Austria (96) also indicates that strict disapproval is not the rule.

As the above-mentioned surveys show as well, very few people do not care about animals, and most think that animals should only be harmed if sufficient benefits are on the horizon. This is reflected in the Directive, which makes harm-benefit analysis one aspect of project evaluation [Directive, Art. 38 (2) d; (68, 80, 97–101)]. With this, the Directive adds an important aspect, asking whether the research aim outweighs the harm (if there is any) on the animal side. This goes well beyond the 3Rs principle, which focuses on how to achieve a given research aim with the least possible animal harm (including no harm at all, in the case of replacement). Hence, in pragmatic terms, current legal requirements (at least in EU Member States) already go beyond the 3Rs principle. That raises questions about whether the principle should be extended, or complemented, and how it feeds into this new responsibility to balance harms and benefits. In any case, the successful interplay of the 3Rs principle and the harm-benefit analysis is in need of a clear methodological understanding of both. Problems of the former have been dealt with in this text; methodological challenges and practical hurdles for carrying out the harm-benefit analysis in a clear and transparent manner have been extensively addressed elsewhere [e.g., (97, 101–104)].

### 5.2. Rethinking replacement strategies

The idea of replacement is typically understood so that animal use is to be avoided if, and only if, the same scientific goal can be achieved with the same quality with alternative methods. Thus, if animals are not *instrumentally necessary* to reach a given research objective, they are replaced by other methods. The correlative question is whether the proposed project, and its use of animals, is appropriate and necessary to achieve a given research objective. However, looking at the idea of replacement more broadly, it can also be asked whether the research objective itself is sufficiently important to justify the harms caused to the animals. This question addresses the *goal-related necessity* of a project – a focus mirrored in the idea of harm-benefit analysis – and opens up a more substantial debate over whether the promised benefits can be reached through means other than the research in question.

These two perspectives – instrumental and goal-related necessity – have been distinguished and elaborated by the Swiss Academy of Science (105). Following the ideas they present, we might say that, to avoid animal use, replacement can also be applied to the way we are trying to obtain prospective benefits. Sometimes non-research alternatives enabling us to obtain benefits without research at all may exist. An example would be reducing the prevalence of a disease by preventive measures rather than the development of treatments. For instance, at least in theory, metabolic syndrome and some cardiovascular problems can be addressed effectively in many patients by dietary changes and exercise. Also in other areas, like production-related diseases in animal farming where animals are kept densely and in big numbers, alternatives might be considered. Rather than finding solutions in new treatment options with the use of biomedical research, changing the production system might efficiently mitigate the problem.

In fact, as a recent qualitative study indicates, the justificatory basis for solving “self-created” problems via animal use in research is weak (96). The study suggests that it is not only the “weight” of benefits that is to be factored into harm-benefit analysis, but also the quality and nature of those benefits. In this regard, the question emerges: What sorts of benefits can actually generate weight on the scales, and what sorts of benefits *cannot* outweigh animal harms and would therefore need to be achieved through means other than research or not at all? Presently, this debate oscillates around the question of benefits achieved in basic research versus those obtained in applied and translational research [for Switzerland *cf.* (106)], but the core of the issue reaches far deeper.

Generally speaking, focusing on research goals allows for debate about what would perhaps be even more efficient, and then non-animal and/or non-research-driven strategies might come into the discussion [e.g., (69)]. This goal-oriented view departs from the original thinking behind the 3Rs, since it questions whether the *replacement of research* (as a strategy to obtain benefits) is an adequate solution to a given problem. Hence, replacement is not only to be understood in terms of replacing animals in research; it can also be understood as taking ethical considerations into account more broadly by asking whether alternative routes bring about comparable benefits with different, and fewer, costs. However, such debates are at an early stage. To ensure they are effective systematic guidance on participation and exchange might be important. For instance, the accessibility and availability of the alternative routes is linked to the different roles that citizens are allocated in discussions of the harms and benefits of animal research, whether as members of the public, participants in research, or potential patients (107).

### 5.3. Approaching reduction and refinement from a different perspective

In the light of ethical considerations, the reduction of animal use can also be reframed. Whereas we should avoid using animals as far as possible – for we are indeed legally bound to do so – there seem to be cases in which animal experiments are still the rule. Sometimes they are even legally required, as they are, for example, in regulatory testing in many – but not all [e.g., U.S. (11)] – countries, as we have seen above. As a matter of fact, the term *reduction* itself (making something *less* in amount or number) already implies the mindset of legitimate animal use for scientific purposes under particular circumstances (e.g., the 3Rs), which conflicts with the idea that animal research should be the exception, rather than the rule [recital 12 Directive; (19)]. Turning this “Yes, but…” into a strict “No, but…” position when we are thinking about possible research aims might help here. This means a shift toward considering animal use in research as an option only in exceptional circumstances, and a corresponding shift of the onus of justification: only if abstaining from the use of animals would produce highly undesirable, unbearable costs, is using them an option. However, the very concept of reduction implies use animals in the first place, and reducing their numbers and the harm done to them to a minimum when designing an experiment – a mindset implicit in the 3Rs principle.

Refinement, and its focus on ameliorating the negative subjective experiences of animals (pain, suffering, distress), has been and might be further reconsidered as well. The centring of refinement strategies on negative subjective experience introduces a risk that we will ignore factors contributing to positive welfare. As far as we know, it is not only pain and suffering that make a difference to an animal’s life. Taking, for example, newer research on cognitive abilities in animals into account, phenomena like the desire to care for conspecifics (108), the preference for stimulating and changing environments (where neophile animals are concerned) with exploratory possibilities (109), certain emotional states, and so on, might enrich the debate and have a bearing on our responsibilities as regards refinement (110, 111). Again, as in the case of replacement and reduction, thinking through the limitations and possible advances here might broaden the debate and allow 3Rs research in the humanities, legal studies and social sciences to be linked with the public debate, and to reflect the issues addressed in a systematic manner. This is particularly important, since all of these ways of broadening our views would occur in an environment where the human-animal relationship is developing.

### 5.4. Conclusions on advancing 3Rs and ethics and society

Where advancement of the 3Rs is concerned, research and insights into the ethical issues, as currently conceived, undertaken in social science, history, law, philosophy, and so forth, can lead to an improved and better-informed understanding of the directions in which we should be moving in the future. Gaining empirical insights into changes of opinion in societies and/or research practices, and reflecting these insights, might allow for more thorough-going development of animal research policies. For instance, comparatively little is known about researchers’ views and opinions. Hence, investigating the epistemology of labs – an epistemology that is often tacit and inherited from one generation to the next – in sociology and anthropology of science [e.g., (112, 113)], could inform the debate substantially. Ideally, the investigation will identify (mental) hurdles linked to attitudes, traditions and management culture [e.g., (61–63, 114, 115)]. Making such matters explicit through systematic investigation might help us to find ways to tackle them. Thus, the humanities and social sciences could offer a richer and fuller picture of the problems that arise when animals are used in research, and in that way support public debate and dialog [e.g., (94, 116, 117)]. It could also take researchers’ views more seriously and treat them indeed as a cornerstone of future efforts to increase our understanding, and improve our use, of the 3Rs. Whether or not this is going to speak in favor of greater efforts being made to protect animals used in research is an open question. In societies where animals are still kept and slaughtered in their billions for food, owned for amusement, hunted and used in sports, it remains unclear which way democratic societies will go.

Looking at the debate on advancing 3Rs from this angle, the humanities and social sciences are not the fifth wheel: they are as important as natural science and biomedicine in the project of advancing implementation of the 3Rs and developing the 3Rs themselves. The proposition that developing humane experimental techniques is both a sociological and scientific problem was present in Russell and Burch’s work in the 1950s, still it fell out of favor as the cultures of science and the humanities diverged. The Swiss National Research Programme “Advancing 3R” (NRP 79) is innovative in its focus on questions about how interdisciplinary research might recover these connections. Research in sociological and scientific disciplines might help to close the gap between science and society. It is to be hoped that it will foster reflective debate on what we can reasonably expect from animal research, and what we are willing to sacrifice for the benefits we hope to gain.

## 6. General discussion and conclusion

The normative principles governing the use of animals are subject to continuous societal negotiation and development. One of the effects of this is that the relationship between us, human beings, and animals can no longer be assumed to be one in which human supremacy reigns. In particular, we can no longer think simply in terms of the instrumental value that animals have for us. To do so, would be to disregard animals’ intrinsic value – a value that is acknowledged in legal documents as well as in common morality. Hence, it is not only in the food production industry, the world of fashion, zoos, our private ownership of pets, and so on, but also in research, that responsible actors have to deal with changing responsibilities and expectations. Given societal change, then, the 3Rs cannot be used as a self-explanatory reference. They need to be continuously re-examined with a view to their normative foundations, their interpretation, their implementation, and their innovation. To give just one illustration of change, we are now much more willing to recognize features such as cognitive and emotional capacities, and prosocial behavior, in non-human species, and to accept the moral responsibilities that follow from such recognition (108, 118–120).

There is a persistent perception that there is a “missing effect” of the implementation of the 3Rs. However, as has been explained in the sections above, proper outcome measures for replacement, reduction and refinement alike are largely undeveloped, and therefore one is entitled to question whether the effect really is missing, or whether instead we are simply unable to properly measure it on a wider, global scale. Total numbers of animals used in research are often the reference point for the public, and for politicians when they point to the supposed missing 3Rs effect. However, when these numbers are not placed in their wider context, what information on the effectiveness of the 3Rs do they provide? A better understanding of the relations between trends and movements in research, in connection with animal use, will not only create an improved understanding of the effects of the 3Rs, but also point to important gaps. So, it is important not only to remove hurdles so as to facilitate 3Rs implementation to the greatest degree possible, but also to develop appropriate outcome measures and understand more fully the environment in which the 3Rs are implemented.

The aims of this paper have been to sketch the potentials, and some success stories, of the 3Rs; to examine the challenges and difficulties of implementing the 3Rs principle; and to identify possible advances in the way the 3Rs are applied and understood. We see a number of ways in which the 3Rs’ effect can be enhanced in the future. First, new or improved tools and methods that increase the probability of successful 3R implementation as well as validation, and broader acceptance of existing tools and methods, will together help to make the 3Rs more effective. Second, strategies to bridge gaps in implementation are of great importance. Third, as we have seen, research on the 3Rs, and particularly on their validation and implementation, generally struggles to find recognition in the funding and publication system. This status problem, as perceived by many scientists, against a background of limited funding incentives, represents an important gap that needs to be filled if we are to encourage greater 3Rs ambition. Hence, fourth, the 3Rs may have more effect if incentives, in the form of reward systems that acknowledge 3R improvements and contribute to career benefits, are put in place. Fifth, to substantiate and develop this further in a bottom-up manner, the inclusion of the 3Rs in training programs and academic curricula is crucial. It will strengthen awareness and knowledge of the 3Rs among scientists. Top-down strategies involving further engagement by regulatory bodies and policy makers in the use of the 3Rs principle as a steering instrument may also encourage the research stakeholder community to advance implementation. Sixth, innovative tools are needed: not just alternative research methods, but tools to raise societal awareness; to promote greater transparency in the research community about animal use; and to encourage dialogue between researchers and ordinary citizens about normative issues raised by the use of animals in research.

In conclusion, the principle of the 3Rs, as an instrument of guidance, is not perfect. Nor is it a comprehensive solution to all of the issues presented by our continuing dependence on animal use in research. To remain useful, the 3Rs therefore need to be both continually advanced at the scientific level and challenged at the normative level. In this way, the goal of responsible scientific knowledge production might be achieved through research involving animals in exceptional cases only.

## Data availability statement

The original contributions presented in the study are included in the article/supplementary material, further inquiries can be directed to the corresponding author.

## Author contributions

HG: conceptualization. HG, IO, JS, and GD: writing—original draft preparation. HG, IO, JS, NB-A, TB, MD, GD, CC, OM, WL, EP, and ET: writing—review and editing. HG, IO, JS, and CC: review and editing final version. All authors have read and agreed to the published version of the manuscript.

## Conflict of interest

While the paper reports on the scientific concept of NRP 79, the opinions and views presented are the authors’ own and do not necessarily represent the position of SNF, the NRP 79 Steering Committee or the authors’ respective institutional affiliations. This paper was developed as an initiative of the Steering Committee of NRP 79. The following members are remunerated for their time invested in NRP 79: NB-A, TB, MD, GD, HG (as president of the SCC), ET, EP, and IO but not for the preparation of this manuscript. The following authors do not receive any remuneration for their engagement in NRP 79 nor in this paper: JS, WL, and OM. This paper reflects the programmatic development of NRP 79 and did not affect project selection.

The remaining author declares that the research was conducted in the absence of any commercial or financial relationships that could be construed as a potential conflict of interest.

## Publisher’s note

All claims expressed in this article are solely those of the authors and do not necessarily represent those of their affiliated organizations, or those of the publisher, the editors and the reviewers. Any product that may be evaluated in this article, or claim that may be made by its manufacturer, is not guaranteed or endorsed by the publisher.
